# AS03-adjuvanted H7N9 inactivated split virion vaccines induce cross-reactive and protective responses in ferrets

**DOI:** 10.1038/s41541-021-00299-3

**Published:** 2021-03-19

**Authors:** Daniel Stadlbauer, Leon de Waal, Edith Beaulieu, Shirin Strohmeier, Edwin J. B. Veldhuis Kroeze, Philippe Boutet, Albert D. M. E. Osterhaus, Florian Krammer, Bruce L. Innis, Raffael Nachbagauer, Koert J. Stittelaar, Corey P. Mallett

**Affiliations:** 1 Department of Microbiology, Icahn School of Medicine at Mount Sinai, New York, NY USA; 2 Viroclinics Biosciences B.V., Viroclinics Xplore, Schaijk, The Netherlands; 3 GSK, Laval, QC Canada; 4 Department of Biotechnology, University of Natural Resources and Life Sciences, Vienna, Austria; 5 GSK, Wavre, Belgium; 6 The Research Center for Emerging Infections and Zoonoses, University of Veterinary Medicine, Hannover, Germany; 7 GSK, King of Prussia, PA USA; 8 Present Address: Nexelis, Laval, QC Canada; 9 Present Address: PATH, Center for Vaccine Innovation and Access, Washington, DC USA; 10 Present Address: Moderna Inc., Cambridge, MA USA; 11 Present Address: Wageningen Bioveterinary Research, Wageningen University & Research, Lelystad, The Netherlands; 12 Present Address: GSK, Rockville, MD USA

**Keywords:** Adaptive immunity, Infectious diseases, Infectious diseases, Influenza virus, Diseases

## Abstract

Human infections with avian H7N9 subtype influenza viruses are a major public health concern and vaccines against H7N9 are urgently needed for pandemic preparedness. In early 2013, novel H7N9 influenza viruses emerged in China that caused about 1600 human cases of infection with a high associated case fatality rate. In this study, two H7N9 split virion vaccines with or without AS03 adjuvant were tested in the naive ferret model. Serological analyses demonstrated that homologous hemagglutination inhibition and microneutralization antibody titers were detectable in the ferrets after the first immunization with the AS03-adjuvanted vaccines that were further boosted by the second immunization. In addition, heterologous antibody titers against older H7 subtype viruses of the North American lineage (H7N7, H7N3) and newer H7 subtype viruses of the Eurasian lineage (H7N9) were detected in the animals receiving the AS03-adjuvanted vaccines. Animals receiving two immunizations of the AS03-adjuvanted vaccines were protected from weight loss and fever in the homologous challenge study and had no detectable virus in throat or lung samples. In addition, microscopic examination post-challenge showed animals immunized with the AS03-adjuvanted vaccines had the least signs of lung injury and inflammation, consistent with the greater relative efficacy of the adjuvanted vaccines. In conclusion, this study demonstrated that the AS03-adjuvanted H7N9 vaccines elicited high levels of homologous and heterologous antibodies and protected against H7N9 virus damage post-challenge.

## Introduction

Influenza viruses infect millions of people worldwide and result in ~290,000–650,000 influenza-related deaths each year^1^. In addition to seasonally occurring human infections, zoonotic infections caused by avian influenza A viruses are a major public health concern and pose a pandemic threat. In 2013, an avian H7N9 virus strain emerged in China that caused hundreds of human infections. From 2013 to 2017, the H7N9 virus led to annual epidemics. During these five waves of epidemics ~1600 laboratory-confirmed cases have been reported, coupled with a case fatality rate of almost 40%^2^. Human infections with H7N9 viruses occurred each year and the viruses gained virulence markers that potentially enhance the risk for humans and may have increased their spread into the human population, making this virus a notable pandemic threat^3,4^. During the fifth wave of H7N9 epidemics the virus split into two phylogenetically distinct lineages, the Yangtze River Delta and Pearl River Delta clades^5^. Ferret antisera raised against existing candidate vaccine viruses (CVVs) showed reduced cross-reactivity against these novel H7N9 viruses^6^. In addition, highly pathogenic avian influenza (HPAI) H7N9 viruses emerged that featured a polybasic cleavage site in the hemagglutinin (HA) and were lethal for poultry^7,8^. However, no community transmission has been reported and infections occurred almost exclusively after exposure to infected poultry. Nevertheless, the large number of human cases increased the likelihood of genetic reassortment of H7N9 viruses with human seasonal influenza viruses, which could lead to sustained human-to-human transmission^9^. In China, cases of people co-infected with both H7N9 and seasonal influenza virus strains have been reported during the period of overlapping seasonal and H7N9 epidemics^10^. In addition, an avian H7N2 virus caused an outbreak in cats in an animal shelter in New York that led to one human case^11^.

Humans are immunologically naive to H7 subtype viruses and possess little to no pre-existing, humoral immunity^12^. Currently, there is no licensed H7N9 vaccine available and people infected with H7N9 viruses are only treated therapeutically with neuraminidase inhibitors. However, H7N9 is quickly acquiring resistance to neuraminidase inhibitors^13^ which is leading to an unreliable public health strategy to combat this virus. To provide more effective protection, vaccines are urgently needed. A phase I/II trial investigating a pre-pandemic H7N9 vaccine, that is also being evaluated in the present study, showed promising results in eliciting robust immune responses in healthy adults, but it is unknown if this vaccine can induce cross-reactive antibody responses against recent fifth wave H7N9 viruses and elicit protective efficacy upon live virus challenge^14^. In this study, H7N9 split virion vaccines, produced at two different GlaxoSmithKline (GSK) production sites (Quebec (Q) and Dresden (D)), are being evaluated. In context of pandemic preparedness, testing of vaccines produced in different regions of world is of importance and contributes to global preparedness efforts.

Ferrets are highly susceptible to influenza virus infection, show clinical symptoms similar to humans and are the preferred animal model to provide evidence of efficacy of influenza virus vaccines especially against highly pathogenic avian influenza viruses^15,16^. Previous preclinical studies in the ferret model have shown that protective immune responses against homologous and heterologous infection with H5N1 viruses can be induced with low doses of H5N1 inactivated split virion vaccines adjuvanted with AS03 (an adjuvant system containing α-tocopherol and squalene in an oil-in-water emulsion). The use of AS03 adjuvant reduced the required dose of HA antigen and resulted in higher and cross-protective immune responses^15,17^. An AS03-adjuvanted H5N1 split virus vaccine (produced at the GSK Quebec site) has been licensed by FDA for human use, however, there is no FDA approved H7N9 vaccine available yet.

The present study was designed to evaluate the potential of AS03-adjuvanted H7N9 A/Shanghai/2/2013 vaccines to induce humoral immunity to a panel of H7 viruses and protection against vaccine-homologous challenge with wild-type H7N9 A/Anhui/1/2013 virus in the naive ferret model.

## Results

### Immunization with AS03-adjuvanted H7N9 split virion vaccines induced homologous and heterologous antibody responses

**Fig. 1:**
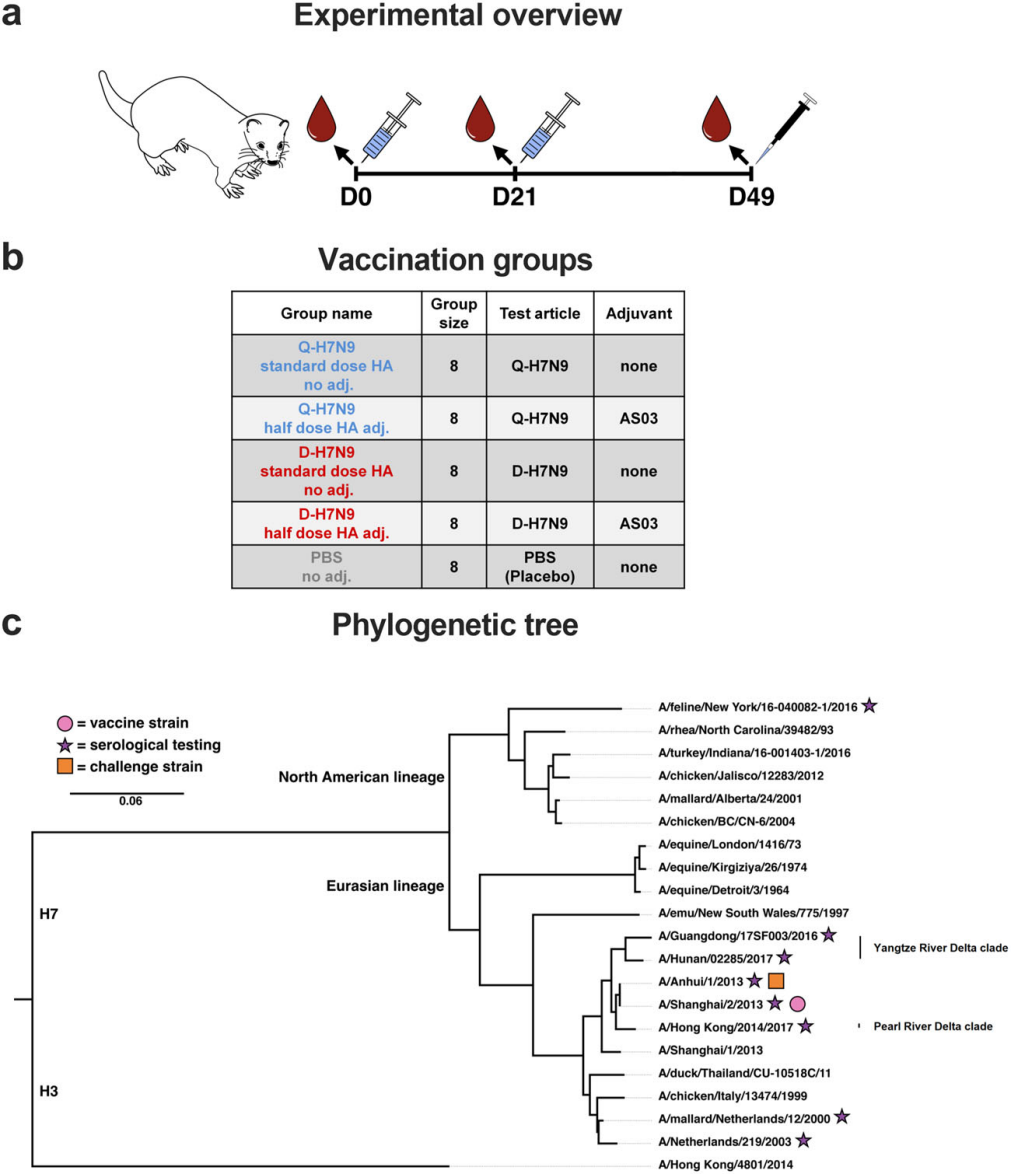
Immunization overview and phylogenetic tree. **a** Experimental overview. Ferrets were immunized on day 0 and day 21 with AS03-adjuvanted or non-adjuvanted H7N9 split virion vaccines. The placebo group received PBS. The animals were bled for serology on days 0, 21, and 49. On day 49, the animals were challenged with a wild-type H7N9 virus. **b** Treatment groups. The five different treatment groups are shown. Two groups (*n* = 8/group) were immunized twice with non-adjuvanted standard-dose Q-H7N9 or D-H7N9 vaccine, respectively. Two groups (*n* = 8/group) were immunized twice with AS03-adjuvanted half-dose Q-H7N9 or D-H7N9 vaccine, respectively, and eight animals were injected with PBS. **c** Phylogenetic tree. North American lineage and Eurasian lineage H7 HAs are shown with H3 HA (A/Hong Kong/4801/2014) as an outgroup. The vaccine strain H7 HA is indicated with a pink closed circle, the H7 strains used for serological testing (HI, VN, ELISA) are highlighted with stars and the H7N9 challenge virus is marked with a square. The scale bar (0.06) indicates the percent difference based on amino acid sequences.

Female ferrets (*n* = 8 per group) were immunized with non-adjuvanted standard-dose H7N9 vaccines or AS03-adjuvanted half-dose H7N9 vaccines twice 21 days apart. Blood for serological testing was taken before the first immunization and after the first (prime) and second (booster) immunizations. The experimental overview, vaccination groups, and virus strains used for vaccination, serological testing, and virus challenge are shown in Fig. 1. The antigenic relationship between the vaccine strain virus and the virus strains used for testing is shown in Supplementary Table 1.

**Fig. 2:**
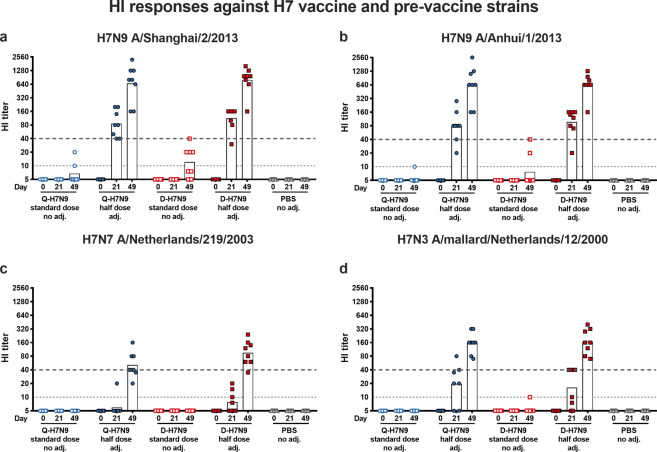
Hemagglutination inhibition (HI) titers against homologous H7N9 viruses and heterologous H7N3 and H7N7 viruses. HI titers against H7N9 A/Shanghai/2/2013 (**a**), H7N9 A/Anhui/1/2013 (**b**), H7N7 A/Netherlands/219/2003 (**c**), and H7N3 A/mallard/Netherlands/12/2000 (**d**) are shown on the *y*-axis. The dashed gray line indicates an HI titer of 1:40. The *x*-axis shows the timepoints (day 0, 21, 49) and the treatment groups are indicated. The dashed gray line at an HI titer of 1:10 represents the limit of detection.

**Fig. 3:**
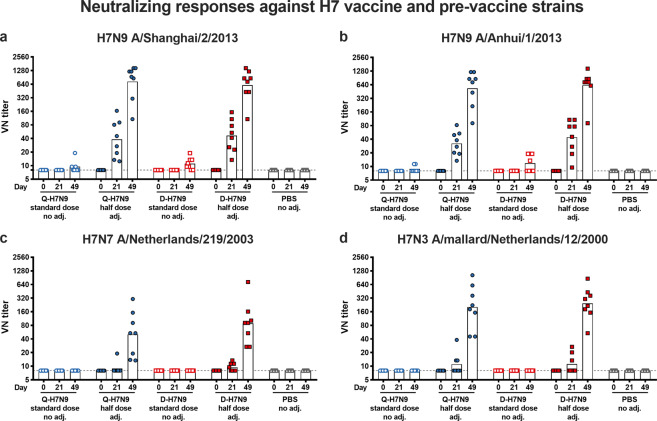
Microneutralization titers (VN) against homologous H7N9 viruses and heterologous H7N3 and H7N7 viruses. VN titers against H7N9 A/Shanghai/2/2013 (**a**), H7N9 A/Anhui/1/2013 (**b**), H7N7 A/Netherlands/219/2003 (**c**), and H7N3 A/mallard/Netherlands/12/2000 (**d**) are shown on the *y*-axis. The *x*-axis shows the timepoints (day 0, 21, 49) and the treatment groups are indicated. The dashed gray lines represent the limit of detection.

Functional antibody titers specific for H7 HA were determined in hemagglutination inhibition (HI; Fig. 2) and virus neutralization assays (VN; Fig. 3) against the homologous H7N9 vaccine strain and antigenically heterologous H7N7 and H7N3 viruses, which circulated prior to the strain selected for the vaccines. On the day of the first immunization (day 0), all ferrets were HI-negative with titers of 5 against the tested viruses. All animals in the non-adjuvanted standard-dose HA groups had non-detectable HI titers after the first immunization at day 21 against the four viruses tested (Fig. 2). After the second vaccination, some animals had detectable HI titers against homologous H7N9 A/Shanghai/2/2013 virus with a reciprocal geometric mean titer (GMT) of 7 in the Q-H7N9 and 12 in the D-H7N9 group. Three animals had detectable HI titers against H7N9 A/Anhui/1/2013, but no HI-active antibodies were induced in the non-adjuvanted standard-dose HA groups against heterologous H7N7 and H7N3 viruses, except for one animal against H7N3 virus after two immunizations in the non-adjuvanted D-H7N9 group (Fig. 2c, d).

In contrast, almost all ferrets primed with the AS03-adjuvanted half-dose HA vaccines seroconverted (as defined by a reciprocal HI titer of 40 or higher) against homologous H7N9 A/Shanghai/2/2013 and A/Anhui/1/2013 virus. Notably, six animals (2/8 in Q-H7N9 and in 4/8 D-H7N9 AS03-adjuvanted half-dose HA group) seroconverted against heterologous H7N3 virus after a single immunization, whereas only five ferrets (1 in Q-H7N9 and 4 in D-H7N9) had a rise in HI antibodies against H7N7 virus. These titers were strongly boosted after the second immunization, reaching high reciprocal GMTs of 666 (Q-H7N9) and 788 (D-H7N9) against A/Shanghai/2/2013 and 629 (Q-H7N9) and 635 (D-H7N9) against A/Anhui/1/2013 H7N9 virus at day 49 post-second immunization (Fig. 2a, b). All animals showed an increase in HI titer against heterologous H7N7 virus, with all but three animals (2 in Q-H7N9 and 1 in D-H7N9 group) seroconverting at day 49 (reciprocal GMT of 51 in Q-H7N9 and 95 in D-H7N9 group). Sera tested against heterologous H7N3 virus showed that all animals seroconverted, resulting in reciprocal GMTs of 157 and 167 for Q-H7N9 and D-H7N9, respectively. Serum samples from the animals receiving PBS placebo were HI-negative against all viruses at all timepoints (Fig. 2a–d).

Next, virus-neutralizing (VN) responses against the same viruses were measured (Fig. 3). A priming immunization with non-adjuvanted standard-dose HA vaccines did not induce VN responses against the viruses, but after a booster immunization, VN responses could be detected against homologous H7N9 viruses in a few ferrets (Fig. 3a, b) with reciprocal GMTs of ~10 (both A/Shanghai/2/2013 and A/Anhui/1/2013). Similar to the HI responses, a priming immunization with AS03-adjuvanted half-dose H7N9 vaccines induced a rise in VN antibodies against H7N9 viruses in all ferrets. These titers increased after the booster immunization, reaching reciprocal GMTs of 731 and 534 for A/Shanghai/2/2013 and A/Anhui/1/2013, respectively, in the animals receiving Q-H7N9. Immunization with AS03-adjuvanted half-dose D-H7N9 boosted the titers to reciprocal GMTs of 608 (A/Shanghai/2/2013) and 622 (A/Anhui/1/2013). None of the PBS placebo-treated ferrets had detectable VN titers. Heterologous neutralizing antibody responses were weaker, resulting in reciprocal GMTs of 51 (Q-H7N9) and 88 (D-H7N9) against H7N7 (A/Netherlands/219/2003) and 203 (Q-H7N9) and 245 (D-H7N9) against H7N7 (A/mallard/Netherlands/12/2000) in the group receiving the AS03-adjuvanted half-dose H7N9 vaccines twice (Fig. 3c, d).

These results support and highlight the beneficial effects of adding the AS03 adjuvant to the H7N9 vaccines in order to increase the immunogenicity of an H7 HA vaccine antigen, to increase the breadth of the functional antibody response, and to enable an antigen-sparing vaccine approach.

### Immunization with AS03-adjuvanted H7N9 split virion vaccines induced antibodies reactive toward fifth wave H7N9 viruses and a North American lineage H7N2 virus

**Fig. 4:**
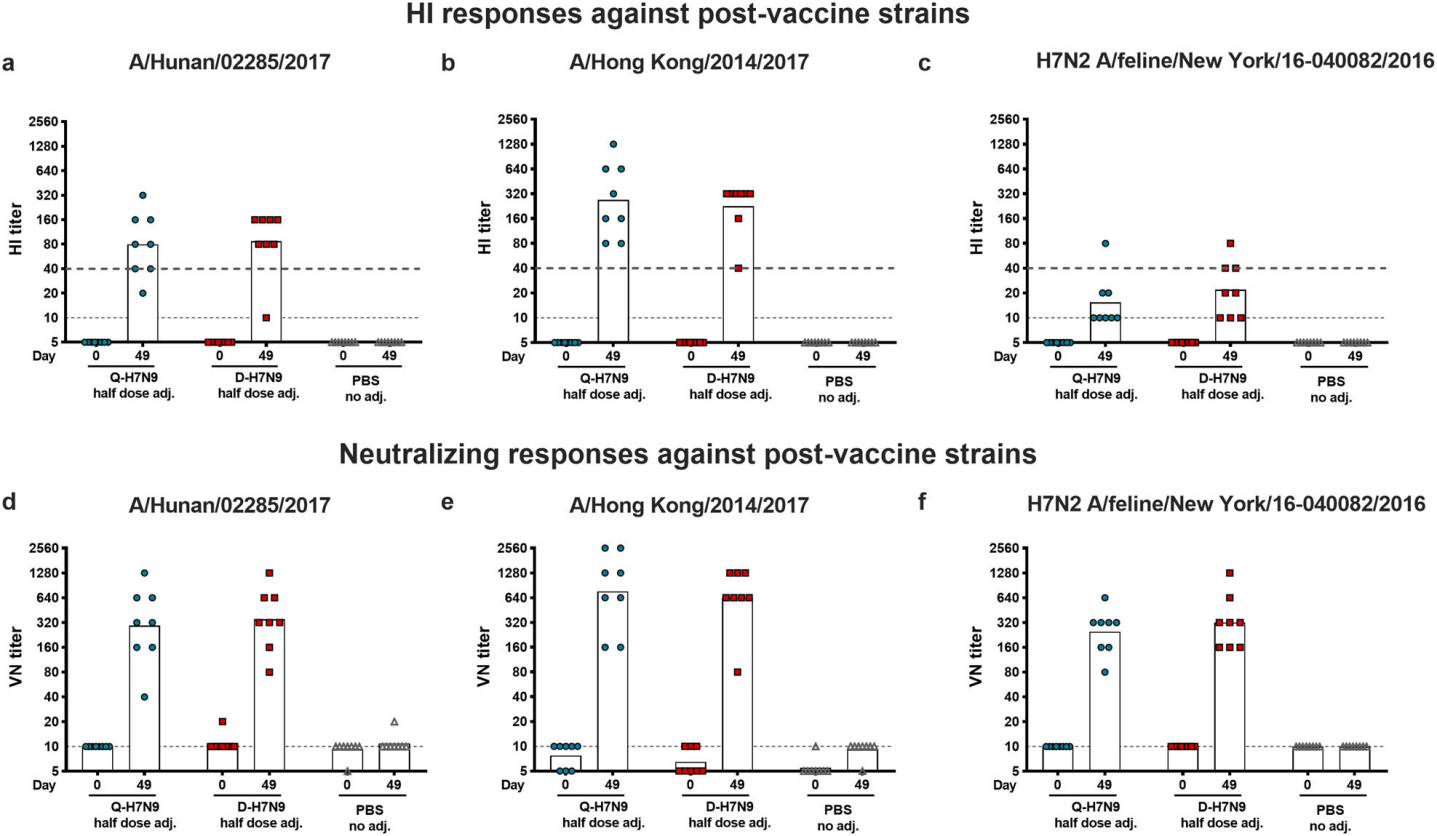
Hemagglutination inhibition (HI) and microneutralization titers (VN) against fifth wave H7N9 viruses and a North American lineage H7N2 virus. The HI titers against H7N1 A/Hunan/02285/2017 (**a**), H7N1 A/Hong Kong/2014/2017 (**b**), and H7N2 A/feline/New York/16-040082/2016 (**c**) are plotted on the *y*-axis. The dashed gray line represents an HI titer of 1:40. The *x*-axis shows the timepoints (day 0, 49) and the treatment groups are indicated. VN responses against the same viruses are shown in **d**–**f**. The *y*-axis shows the VN titer, the timepoints (day 0, 49) and treatment groups are indicated. The dashed gray line at an HI or VN titer of 1:10 indicates the limit of detection.

Cross-reactive antibody responses were further investigated in the ferrets receiving the adjuvanted vaccines (half-dose HA + AS03) (Fig. 4). For the HI and VN assays, two fifth wave H7N9 viruses, including one representative virus from each of the new fifth wave clade (Pearl River Delta clade, Yangtze River Delta clade), and a feline H7N2 virus were used^6,11^. These viruses circulated more recently than the virus strain used in the vaccine and are antigenically distinct from the vaccine strain virus (Supplementary Table 1). In addition, the feline virus is from a different genetic HA lineage than the vaccine strain. All animals receiving the AS03-adjuvanted H7N9 vaccines developed HI and VN antibody titers against fifth wave H7 viruses (Fig. 4a, b, d, e). At day 49, the animals receiving the Q-H7N9 vaccine developed a reciprocal HI GMT of 80 and a reciprocal VN GMT of 293 against A/Hunan/02285/2017 (Yangtze River Delta clade) as well as a reciprocal HI GMT of 269 and a reciprocal VN GMT of 761 against A/Hong Kong/2014/2017 (Pearl River Delta clade). The ferrets vaccinated with the AS03-adjuvanted half-dose D-H7N9 vaccine developed similar reciprocal HI GMTs of 87 and reciprocal VN GMT of 349 against A/Hunan/02285/2017 and reciprocal HI GMT of 226 and reciprocal VN GMT of 640 against A/Hong Kong/2014/2017.

To test if immunization with a Eurasian lineage H7N9 virus vaccine can also induce antibodies cross-reactive toward a North American lineage H7 virus, we tested a feline H7N2 virus in HI and VN assays (Fig. 4c, f). Immunization with AS03-adjuvanted H7N9 vaccines induced low HI titers with a reciprocal GMT of 15 (Q-H7N9) and 22 (D-H7N9) against H7N2 A/feline/New York/16-040082/2016, but higher reciprocal VN GMTs of 247 (Q-H7N9) and 320 (D-H7N9) against H7N2 A/feline/New York/16-040082/2016, indicating that H7 cross-neutralizing antibodies were generated. Interestingly, we found a larger difference between HI and VN titers for the feline virus, whereas the HI and VN titers for the other viruses tested were rather similar, which might be attributable to different affinities of the viruses to the red blood cells used in this assay.

To investigate cross-reactive antibody responses in more detail, IgG binding antibodies were measured by ELISA in the animals immunized with AS03-adjuvanted half-dose H7N9 split virion vaccines, which showed robust binding against all tested HAs from the panel of H7 viruses (Supplementary Fig. 1).

### Immunized ferrets were protected against H7N9 virus challenge

**Fig. 5:**
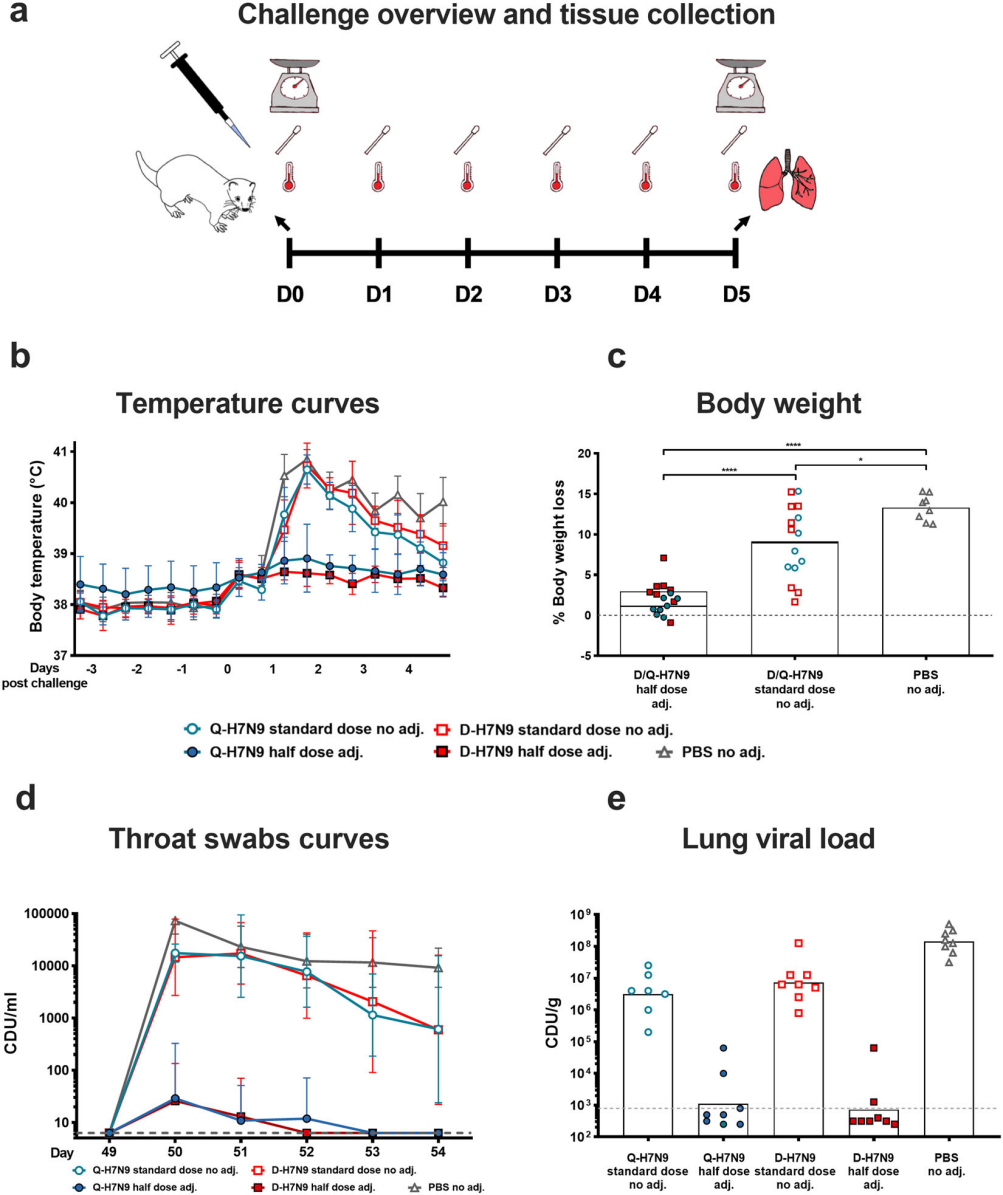
Challenge experiment overview, morbidity, and viral titers. **a** Experimental overview. On day 0 (day 49 post-prime) ferret body temperatures and body weights were measured, and throat and nose swabs taken. The body temperatures were monitored continuously with a 10 min interval until the scheduled day of euthanasia (day 5 post-challenge) and nose and throat swabs were collected daily. On day 5 the body weights were recorded, and the animals were euthanized. Nasal turbinates and lungs were collected for virus quantification and histopathology. **b** Temperature curves. Body temperature curves (in °C) starting three days before virus challenge until the day of euthanasia (day 5 post-challenge) are shown for the animals receiving non-adjuvanted standard-dose H7N9 split virion vaccines (open circles, open squares), AS03-adjuvanted half-dose H7N9 split virion vaccines (closed circle, closed squares) or PBS (open triangle). The geometric mean of the body temperature of the ferrets per group and geometric standard deviation are shown. **c** Body weight loss. The body weight at the day of challenge (day 0) and day of euthanasia (day 5) were recorded and the weight loss (in %) plotted on the *y*-axis. The half-dose, standard dose, and PBS groups were compared in a one-way ANOVA Tukey’s multiple comparisons test, *P < 0.0332, ****P < 0.0001. **d** Throat swab curves. Quantification of viral RNA by PCR expressed as CDU/mL is depicted for the five different groups (day 0–day 5). The geometric mean of the CDU/mL and geometric standard deviation are shown. **e** Lung viral load. Lung viral titers recorded as CDU/g lung tissue is plotted on the *y*-axis. All samples with a value smaller than the value indicated by the dashed gray line fall below the limit of detection.

To investigate the protective efficacy of the H7N9 split virion vaccines, the animals were challenged with a sub-lethal dose of wild-type H7N9 (A/Anhui/1/2013) virus on day 49. The body temperatures were monitored continuously, and nose and throat swabs were collected daily. Body weights were recorded at challenge and on the day of scheduled euthanasia (day 5 post-challenge). The lungs and nasal turbinates were also harvested on the day of scheduled euthanasia (Fig. 5a). The animals receiving the non-adjuvanted Q-H7N9 or D-H7N9 vaccines (standard dose) showed a high mean body temperature starting from 24 to 48 h after challenge (Fig. 5b). In the AS03-adjuvanted H7N9 groups (half-dose) the ferrets showed a slightly elevated mean body temperature after challenge but did not develop fever. The animals in the PBS group had high mean body temperature and fever starting around 24 h after challenge.

The animals receiving the AS03-adjuvanted half-dose H7N9 vaccines were protected against morbidity in terms of little to no body weight loss post-challenge with a mean body weight loss of 1% (Q-H7N9) or 3% (D-H7N9). Significantly higher body weight loss was observed in ferrets receiving non-adjuvanted standard-dose vaccines (9% mean body weight loss, both Q- and D-H7N9) or PBS placebo (13% mean body weight loss) (Fig. 5c).

To investigate viral replication in the upper respiratory tract, nose and throat swabs were collected daily post-challenge and levels of virus shedding measured by PCR and/or virus titration on MDCK cells. The ferrets immunized with non-adjuvanted standard-dose H7N9 vaccines or injected with PBS placebo showed high levels of viral RNA in the throat after challenge, whereas immunization with the AS03-adjuvanted half-dose H7N9 vaccines reduced the amount of detectable viral RNA in the throat region by at least 100-fold (Fig. 5d). Samples that had detectable viral RNA were further tested by performing virus titration on MDCK cells (Supplementary Fig. 2) and similar results were obtained. No detectable viral RNA was measured in the nose swabs in any of the groups (Supplementary Fig. 3), except in one animal in both the non-adjuvanted H7N9 standard-dose HA group (Q-H7N9) and in one control (PBS placebo) animal.

On day 5 post-challenge, the animals were euthanized, and lung and nasal turbinates were collected to determine viral load. All but two animals (6/8) in the non-adjuvanted standard-dose Q-H7N9 group had replication-competent virus in lung tissues with a geometric mean of 126 TCID_50_/g lung tissue and all animals (8/8) in the non-adjuvanted standard-dose D-H7N9 group had detectable virus with a GM of 972 TCID_50_/g (Supplementary Fig 4). Ferrets immunized with AS03-adjuvanted half-dose H7N9 vaccines had no detectable replication-competent virus in the lung and only four animals (2 in Q-H7N9 group, 2 in D-H7N9) had detectable levels of viral RNA in the lung (Fig. 5e). The viral load in the animals receiving PBS placebo was about twenty times higher (GMT of 1.41 × 10^8^ CDU/g) than animals receiving the standard-dose vaccines (Fig. 5e). Virus titration of the nasal turbinates further confirmed the protective effect of the AS03-adjuvanted half-dose vaccines (Supplementary Fig 5). None of the ferrets in the groups that received the AS03-adjuvanted half-dose vaccines had detectable virus in the nasal turbinates.

These data taken together demonstrate that immunization with AS03-adjuvanted half-dose H7N9 split virion vaccines confer H7 cross-reactive antibody responses and protective immunity against wild-type H7N9 virus infection in the ferret model.

### Histopathological analysis of respiratory tract tissues post-virus challenge

The effect of live virus challenge on the immunized animals was investigated in more detail by performing gross and histopathological evaluation on the trachea and lungs. There was no evidence from the post-mortem examination that the H7N9 influenza virus challenge was confounded by any other infecting microorganisms or underlying disease that was present at the start of the study or acquired during the course of the study.

The intratracheal challenge of ferrets with H7N9 influenza virus led to rhinitis across the treatment groups, although approximately one-third of the animals in the study had no rhinitis at all. On average, animals from one of the AS03-adjuvanted half-dose vaccine groups (D-H7N9) and animals from the PBS placebo group were most severely affected (Table 1). Therefore, the severity of the rhinitis did not correlate well with treatment assignment, and the immunological significance of upper respiratory tract histopathology such as that seen in the nasal turbinates is not well understood in context of the vaccine modality described in this study using a stringent model of lower respiratory tract infection following intratracheal challenge.

**Table 1:** Macroscopic and microscopic lung examination at scheduled euthanasia five days post-H7N9 virus challenge^a^.

Treatment^b^	Estimated % of affected lung		Relative lung weight^c^		Extent of alveolitis/alveolar damage^d^	Severity of alveolitis^d^	Alveolar edema^e^	Alveolar hemorrhage^e^	Type II pneumocyte hyperplasia^e^	Severity of bronchitis/bronchiolitis^d^	Severity of rhinitis^d^
	Mean	Range	Mean	Range							
Q-H7N9 half-dose HA adj.	9	0–10	0.75	0.51–1.42	0.47	0.47	3	0	34	0.25	0.50
Q-H7N9 standard dose HA no adj.	21	10–50	1.21	0.99–1.45	1.57	2.29	93	14	100	1.82	0.57
D-H7N9 half-dose HA adj.	11	0–30	0.71	0.58–1.13	0.47	0.63	6	0	25	0.44	1.13
D-H7N9 standard dose HA no adj.	20	10–50	1.02	0.75–1.44	1.25	1.84	69	25	88	1.59	0.50
PBS no adj.	55	30–90	1.42	1.11–1.96	2.28	2.18	97	53	100	1.91	1.00

^a^Animals challenged with 105.5 TCID50 A/Anhui/1/2013 virus by the IT route.

^b^Animals immunized with AS03-adjuvanted half-dose vaccine or non-adjuvanted standard-dose vaccine. Q indicates vaccine manufactured at the Quebec, Canada facility and D indicates vaccine manufactured at the Dresden, Germany facility.

^c^Relative lung weight equals (lung weight/body weight on day of euthanasia) × 100.

^d^Extent of alveolitis/alveolar damage, severity of alveolitis, severity of bronchitis/bronchiolitis, and severity of rhinitis were based on a score of 0–3.

^e^Alveolar edema, alveolar hemorrhage, and type II pneumocyte hyperplasia were based on the percentage of slides positive.

The intratracheal challenge of ferrets with H7N9 influenza virus resulted in alveolitis/alveolar damage, alveolar edema, and hemorrhage that ranged from mild to severe. The animals that received the AS03-adjuvanted half-dose vaccines were least affected in this location (Table 1). Conversely, the animals that received the non-adjuvanted standard-dose vaccines and the PBS placebo animals all had severe histopathological scores in the alveoli (Table 1). For other parameters assessed such as percent affected lung tissue based on gross visual inspection and relative lung weights, as well as additional histopathological parameters such as bronchitis/bronchiolitis and type II pneumocyte hyperplasia, a very similar pattern was observed in that the severity index correlated well with treatment assignments and the AS03-adjuvanted half-dose H7N9 vaccines achieved the best outcomes (Table 1).

In summary, the histopathological observations in affected pulmonary tissues of ferrets following intratracheal challenge of wild-type H7N9 influenza virus (A/Anhui/1/2013) seen in the current study are consistent with those reported previously in the setup of this preclinical model^18^. In addition, the animals that received the AS03-adjuvanted half-dose vaccines (Table 1) had the best histopathological outcomes which further demonstrated a beneficial AS03 adjuvant effect.

## Discussion

Avian influenza A viruses are a major public health threat. Spillover events of animal viruses into the human population have the potential to cause epidemics or, in the worst case, pandemics^19^. In 2013, an avian influenza H7N9 virus emerged in China that caused five consecutive, annual waves of infections^8,20^. The virus caused infections in humans that had close contact with infected animals, but it did not gain the ability for sustained human-to-human transmission. Nevertheless, vaccines against the H7N9 virus are urgently needed as countermeasures against possible outbreaks and for pandemic preparedness^3^. Humans have no pre-existing, humoral immunity to H7 subtype viruses and it has become clear in earlier studies that an effective adjuvant and/or two-dose vaccination are necessary to achieve protective antibody levels^21–23^.

In the present study, AS03-adjuvanted and non-adjuvanted H7N9 inactivated split virion vaccines administered twice (second injection administered 3 weeks after first injection) were evaluated in the naive ferret model and the protective efficacy tested in a non-lethal challenge study. The present study demonstrated that a half-dose of H7N9 inactivated split virion vaccines induced a potent humoral immune response in ferrets when formulated with an effective adjuvant such as AS03. A standard dose of non-adjuvanted vaccines was unable to induce a high percentage of HI seroconversion, highlighting the benefit of the adjuvant. These findings are consistent with results reported in preclinical studies testing an AS03-adjuvanted H5N1 split virion vaccine in ferrets. The addition of the AS03 adjuvant allowed for dose-sparing and elicited robust antibody responses^15,17^.

Furthermore, two administrations of AS03-adjuvanted half-dose H7N9 vaccines induced cross-reactive antibodies that inhibit hemagglutination by Eurasian lineage H7 viruses (H7N7, H7N3) and neutralized the same viruses in vitro. In addition, vaccination with the AS03-adjuvanted half-dose vaccines elicited HI and neutralizing antibodies that reacted against fifth wave H7N9 viruses as well as a feline H7N2 virus of the North American lineage. These results indicate that an AS03-adjuvanted HA vaccine antigen derived from a first wave virus can induce cross-reactive antibodies reacting to more recent fifth wave viruses as well as to other H7 viruses. This cross-reactivity is likely based on conserved shared epitopes, either on the H7 HA head or HA stalk domain, that are not subjected to immune pressure^23,24^. During the fifth epidemic wave, H7N9 viruses showed more genetic diversity, but recent structural studies report that fifth wave H7 HAs have similar overall structures as compared to a first wave H7 HA. However, based on several amino acid changes in antigenic site A of LPAI H7 HA and the occurrence of HPAI viruses with changes in HA antigenic sites A, C, and D, new candidate vaccine viruses have been proposed to cover these changes^25^.

A previously established low pathogenic avian influenza A H7N9 virus challenge model in ferrets was adapted and employed to test the protective efficacy of the H7N9 split virion vaccine^18^. The viral challenge experiment demonstrated that the adjuvanted vaccines can suppress fever, minimize body weight loss, and reduce the viral load in throat swabs and lungs (Fig. 5). A more detailed histopathological examination of various tissues showed that the AS03-adjuvanted H7N9 vaccines provided superior protection against lung tissue injury subsequent to viral challenge further demonstrating the beneficial AS03 adjuvant effect (Table 1). In addition, the AS03-adjuvanted half-dose H7N9 vaccines reduced viral titers in the upper respiratory tract (throat and nose) which can lead to reduced viral transmission.

Other H7N9 vaccine candidates have been tested in preclinical studies and have shown to be immunogenic and protective in animal models. These vaccines include H7N9 split vaccines using different adjuvants like SWE (an oil-in-water adjuvant)^26^ and MF59^27,28^ or non-adjuvanted split vaccines^29^. Other approaches are based on live attenuated virus vaccines^30,31^, recombinant H7 HA formulations^32^, or mRNA^33,34^. The AS03-adjuvanted H7N9 split virion vaccines described in this report compare favorably or, in some cases, out-perform these other vaccine candidates. Favorable results in preclinical studies led to the initiation of human clinical trials testing these pre-pandemic H7N9 split vaccines in human adults^14^ and are an important component of pandemic preparedness efforts. The present study provides important data that demonstrates induction of cross-reactive antibody responses upon vaccination, which was not investigated and shown prior. These results inform pandemic preparedness strategies moving forward and could help advance other vaccine candidates into clinical trials and provides promising data to continue clinical development.

In conclusion, the present study demonstrated that H7N9 split virion vaccines adjuvanted with AS03 induced broad and potent antibody responses in naive ferrets and protected against homologous viral challenge with low pathogenic H7N9 virus. These preclinical results together with previously reported phase I/II results support the advanced clinical development of the AS03-adjuvanted H7N9 split virion vaccines.

## Methods

### Vaccines and AS03 adjuvant system

The investigational monovalent H7N9 egg-derived, inactivated, detergent-split virion vaccines used in this study were manufactured from A/Shanghai/2/2013 (H7N9)-PR8-IDCDC-RG32A reassortant vaccine virus (Centers for Disease Control and Prevention, Atlanta, GA, USA) using reverse genetics; the virus contains the HA and NA genes of low pathogenic avian A/Shanghai/2/2013 (H7N9) virus plus six genes from A/Puerto Rico/8/1934 (H1N1) virus. The sequence is based on a virus initially isolated from a patient with fatal disease. The vaccine was manufactured at two different GSK production sites, Quebec (Q-H7N9) and Dresden (D-H7N9). The Q-H7N9 vaccine was manufactured at GSK, Sainte-Foy, Québec, Canada, using the FluLaval Quadrivalent seasonal influenza vaccine process, whereas the D-H7N9 vaccine was manufactured at GSK, Dresden, Germany, using the Fluarix Quadrivalent seasonal influenza vaccine process slightly modified. The Q-H7N9 vaccine was further developed and tested in a human clinical trial^14^. Both vaccines were used for the ferret immunizations as final bulk material manufactured under good manufacturing practice (GMP). AS03^35^ was manufactured under good manufacturing practice (GMP) by GSK. AS03 is an Adjuvant System containing α-tocopherol and squalene in an oil-in-water emulsion (11.86 mg α-tocopherol). The AS03 used for the study described herein is the equivalent of the adult human dose. The detergent-split virion vaccines were ad-mixed with the AS03 by gentle inversion preceding each immunization.

The standard-dose non-adjuvanted vaccine formulation consisted of a target dose of 15 µg HA, whereas the half-dose adjuvanted formulations consisted of a target dose of 7.5 µg HA mixed at a 1:1 ratio with AS03 adjuvant containing 47.44 mg/ml α-tocopherol. Standard and half-dose formulations refer to standard or half doses of split virion influenza vaccines typically given to human adults. The HA content for formulation was measured by single radial immunodiffusion (SRID) assay. The antigen dose for the Q-H7N9 vaccines were less than the initially intended concentrations of 15 and 7.5 µg HA because the SRID assay used to measure the antigen concentration overestimated the antigen concentration in relation to later available reagents provided by the Center for Biologics Evaluation and Research (CBER). The adjusted vaccine doses for the Q-H7N9 vaccines based on revised SRID values were 10.5 µg HA in the non-adjuvanted standard-dose vaccine and 5.25 µg HA in the half-dose adjuvanted vaccine. Both vaccine formulations contained 0.001% thimerosal. Sterile phosphate-buffered saline (PBS; Viroclinics Biosciences B.V.) was given to the placebo group. The vaccine formulations were prepared on the day of administration (day 0 and day 21).

### Animals and husbandry

The animal study was carried out in the central animal facilities of The Netherlands Vaccine Institute (NVI; Bilthoven, The Netherlands). The NVI is approved for animal work under local legislation; Dutch Animal Experimentation Act, 1977 (Wet op de dierproeven, 1977), which is the Dutch equivalent to the European Council Directive 86/609 EEG. The protocol was licensed under protocol number 201300081 by the animal ethics committee. The study was conducted in accordance with the GSK Policy on the Care, Welfare, and Treatment of Laboratory Animals and it was reviewed by the Institutional Animal Care and Use Committee at the NVI.

The ferrets were serologically tested for Aleutian disease using counterimmunoelectrophoresis (CIEP) and were confirmed seronegative for circulating H1N1 and H3N2 virus as well as H7N9 (A/Anhui/1/2013) virus in hemagglutination inhibition assays. Only animals that were found seronegative for Aleutian disease and influenza viruses were used for the current study. The animals were housed in normal cages in groups of eight animals during the acclimation phase and immunization phase, and they were transferred to glovebox isolator cages on the day of the challenge. The animals had free access to food and water. For all experimental manipulations, the ferrets were anesthetized by intramuscular injection with ketamine or a mixture of ketamine and medetomidine.

### Ferret vaccination and intratracheal challenge

Eight female ferrets were used per vaccine or PBS placebo group. Animals were immunized on days 0 and 21 with non-adjuvanted standard-dose H7N9 split virion Q-H7N9 or D-H7N9 vaccine, AS03-adjuvanted half-dose H7N9 split virion Q-H7N9 or D-H7N9, or PBS placebo. On days 0, 21, and 49, 2.5 mL of blood was collected from the jugular vein in clot tubes from anesthetized animals. The tubes were centrifuged at 2000 × *g* for 10 min at room temperature, serum was collected, and stored at −80 °C until used for serology. The immunization scheme and overview of the study groups can be seen in Fig. 1a, b. Body weight was monitored on days 0, 21, 49, and 54. On day 49, the animals were challenged intratracheally with 10^5.5^ tissue culture infectious dose 50 (TCID_50_) wild-type H7N9 (A/Anhui/1/2013) virus under biosafety level 3 containment as previously established^18^. Throat and nose swabs were taken on days 49 through 54, inclusive. Body temperatures were monitored continuously with a 10 min interval by means of an implanted recorder (Star-Oddi, Gardabaer, Iceland). The recorder was implanted into the peritoneal cavity 36 days prior the first immunization. The animals were euthanized on day 54 and lung tissue and nasal turbinates collected for viral load determination and histopathology.

### Cells, proteins, and viruses

Madin Darby canine kidney (MDCK) and human embryonic kidney (293T) cells were maintained in Dulbecco’s modified Eagle’s medium (DMEM; Gibco) supplemented with penicillin-streptomycin antibiotic mix (100 U/mL of penicillin, 100 μg/mL streptomycin; Gibco) and fetal bovine serum (FBS, 10%; HyClone). BTI-TN5B1-4 (Trichoplusia ni) cells were grown in serum-free SFX medium (HyClone), containing antibiotics (100 U/mL of penicillin, 100 μg/mL streptomycin; Gibco).

The recombinant proteins, including H7 from A/Anhui/1/2013, H7 from A/Guangdong/17SF003/2016, H7 from A/Hunan/02285/2017, H7 from A/Hong Kong/2014/2017, and H7 from A/feline/New York/16-040082/2016 were produced in the baculovirus expression vector system as previously described^36,37^. The polybasic cleavage site of the highly pathogenic avian isolate A/Guangdong/17SF003/2016 was removed, resulting in a low pathogenic avian influenza H7N9 cleavage site and increased protein stability.

The H7 low pathogenic virus variants were rescued in a A/Puerto Rico/8/1934 (PR8) background by reverse genetics techniques as previously described^38^. Briefly, the H7 HA segment of A/Hong Kong/2014/2017 and A/Hunan/02285/2017 were combined, respectively, with the seven other genomic influenza A segments of PR8, resulting in 7:1 reassortants. The H7 HA and N2 NA segment of A/feline/New York/16-040082/2016 were combined with the six internal segments of PR8, resulting in 6:2 virus variants. The HA and NA cDNA was synthetically produced (Thermo Fisher), and all virus reassortants were sequenced to confirm the genotype. The viruses were grown in 8–10-day-old embryonated chicken eggs (Charles River Laboratories) at 37 °C for 48 h and the allantoic fluid was harvested.

The wild-type influenza H7N9 A/Anhui/1/2013 virus isolate used for the challenge phase was passaged three times in embryonated chicken eggs and once in MDCK cells. The virus is based on an isolate from a fatal human case in China and was kindly provided by the Pandemic Influenza Preparedness Framework^18^.

Viruses and proteins used in serological assays in this study are indicated in the phylogenetic tree in Fig. 1c.

### Enzyme-linked immunosorbent assay (ELISA)

Microtiter plates (96-well, Thermo Fisher) were coated with recombinant H7 HA proteins at a concentration of 2 µg/mL diluted in coating buffer (SeraCare) at 4 °C overnight. The following day, the plates were washed three times with PBS (Gibco) supplemented with 0.1% Tween 20 (PBS-T) and blocked with 220 µL of blocking solution (PBS-T supplemented with 3% goat serum (Life Technologies) and 0.5% milk powder (American Bio) for 1 h at room temperature. Ferret serum samples were diluted to an initial starting concentration of 1:100, serially diluted 1:2 in blocking solution, and incubated for 2 h at room temperature. The plates were washed three times with PBS-T and 50 µL anti-ferret IgG (goat anti-ferret IgG (gamma chain-specific) horseradish peroxidase (HRP) conjugate, Alpha Diagnostics, #70530) per well was added. The 96-well plates were incubated for 1 h at room temperature, washed four times with PBS-T, and developed with 100 µL/well of SigmaFast o-phenylenediamine dihydrochloride (OPD; Sigma). To stop the reaction, 50 µL/well 3 M hydrochloric acid (Thermo Fisher) was added and the plates were read at 490 nm with a microtiter plate reader (BioTek). The data were analyzed in Microsoft Excel and GraphPad Prism Version 8. The cutoff value was defined as the average of all blank wells plus three times the standard deviation of the blanks. The area under the curve (AUC) was calculated based on the cutoff value.

### Hemagglutination inhibition

Hemagglutination inhibition (HI) antibody titers against H7N9 A/Shanghai/2/2013, H7N9 A/Anhui/1/2013, H7N7 A/Netherlands/219/2003, and H7N3 A/mallard/Netherlands/12/2000 were determined at Viroclinics as described previoulsy^39^. In brief, after treatment with cholera filtrate (receptor destroying enzyme, RDE) and heat-inactivation at 56 °C, the sera were tested for the presence of anti-HA antibodies. For this purpose, 1% turkey erythrocytes were used and four HA-units of the different H7 influenza strains. Titers of 1:5 were assigned to HI-negative samples to facilitate data analysis and data representation.

Serum antibody titers against the fifth wave viruses (A/Hunan/02285/2017, A/Hong Kong/2014/2017) and H7N2 (A/feline/New York/16-040082/2016) were measured in a slightly modified HI assay at the Icahn School of Medicine at Mount Sinai. The ferret sera were treated with RDE (Denka Seiken) to remove nonspecific inhibition of hemagglutination. In brief, for 25 µL sera 75 µL RDE was added and incubated overnight at 37 °C. The reaction was stopped by the addition of 75 µL sodium citrate (2.5%) and incubation at 56 °C for 1 h. The sera were diluted with 75 µL PBS, resulting in an overall dilution of 1:10 (limit of detection of the assay). The pre-diluted sera were serially diluted 1:2 in v-bottom 96-well plates (Thermo Fisher). The viruses (A/Hunan/02285/2017, A/feline/New York/16-040082/2016, A/Hong Kong/2014/2017) were diluted to 8 hemagglutination units (HAU)/50 µL in PBS and 25 µL/well of virus was added to the serially diluted sera in the v-bottom plates (Sigma). The plates were incubated for 30 min at room temperature on a shaker. Chicken red blood cells (RBCs, Lampire Biologicals) were diluted to a concentration of 0.5% in PBS and 50 µL/well were added to the sera/virus mixture. The plates were incubated at 4 °C for 1 h, the results recorded in Microsoft Excel and analyzed in GraphPad Prism Version 8. The HI titer was defined as the last dilution in which hemagglutination does not occur. Negative samples (hemagglutination at all dilutions) were assigned a value of 5 (half of the limit of detection) for GMT calculation and data representation.

### Microneutralization

Virus neutralization (VN) titers against H7N9 (A/Shanghai/2/2013 and A/Anhui/1/2013), H7N7, and H7N3 were measured at Viroclinics as described previously^39^. In brief, sera were tested after heat-inactivation at 56 °C for 30 min for the presence of VN antibodies using a microneutralization assay with ~100× TCID_50_ of the different H7 influenza strains. Neutralization titers were calculated as the dilution giving 50% neutralization and expressed as the reciprocal of the dilution. Samples below the limit of detection were assigned a value of 8 for GMT calculations and data representation.

The more recently circulating H7 viruses were tested in a modified microneutralization assay at the Icahn School of Medicine at Mount Sinai. MDCK cells (100 µL/well) were seeded at a concentration of 1.6 × 10^5^ cells/mL complete DMEM in 96-well tissue culture plates (Sigma) and incubated overnight at 37 °C. Ferret sera were RDE-treated as described above, resulting in an initial dilution of 1:10 (limit of detection). The pre-diluted sera were serially diluted 1:2 in UltraMDCK media (Lonza), containing tosyl phenylalanyl chloromethyl ketone (TPCK)-treated trypsin (infection media; Sigma) at a concentration of 1 μg/mL, in 96-well culture plates. The viruses were diluted to a concentration of 100-TCID_50_, added to the serially diluted sera, and incubated for 1 h at room temperature on a shaker. The MDCK cells were washed with 220 µL PBS/well and 100 µL/well of the virus/sera mixture was added to the cells. The plates were incubated for 72 h at 33 °C. Chicken RBCs were diluted to a concentration of 0.5% in PBS and 50 µL of cell supernatant was transferred to v-bottom plates (Sigma). RBCs (50 µL/well) were added to the v-bottom plates and the plates were incubated for 1 h at 4 °C. The neutralization titer was defined as the last dilution in which hemagglutination does not occur. Negative samples were assigned a value of 5 (half of the limit of detection) for GMT calculation and data representation. The results were recorded in Microsoft Excel and analyzed in GraphPad Prism Version 8.

### Virus shedding in the upper respiratory tract and viral load in tissues

Levels of viral RNA (from infectious and non-infectious virus) were determined by means of influenza virus A matrix gene-specific TaqMan polymerase chain reaction (PCR) and levels of infectious (replication-competent) virus were determined by virus titration on MDCK cells. Virus RNA load and infectious virus titer in the upper respiratory tract (URT) were expressed as Control Dilution Units (CDU)/mL and TCID_50_/mL, respectively. CDU were determined from a standard curve produced from a stock virus with a known titer which was serially diluted in a 10-fold dilution series, with each dilution undergoing nucleic acid extraction and Taqman PCR amplification in the same manner as test samples. The undiluted virus (virus stock) had a titer of 8.5 log_10_ TCID_50_/mL, was diluted from 10^0^ to 10^−8^ (in 10-fold dilution steps) and tested in triplicate in the PCR resulting in a slope of the standard curve of 3.49 and intercept of 40.84. On the basis of the standard curve, the lower limit of quantification (LLOQ) of PCR was determined to be 0.8 log_10_ CDU/mL. Samples below the LLOQ are shown as <0.8 log_10_ CDU/mL. All samples that gave a Ct value were analyzed by virus titration. Virus titration was performed on a newly thawed aliquot as described elsewhere^40^. The LLOQ of virus titration was 0.8 log_10_ TCID_50_/mL. Samples below this cutoff value were scored as <0.8 log_10_ TCID_50_/mL. Virus RNA load and infectious virus titer in tissues were expressed as log_10_ CDU/gram of tissue and log_10_ TCID_50_/g of tissue, respectively. All tissue samples were analyzed by TaqMan PCR and virus titration. The LLOQ of each lung and turbinate sample varied according to the individual tissue weight.

### Gross and histopathological evaluation

At the time of scheduled euthanasia on day 54, a complete macroscopic gross-pathological assessment of all organ systems was performed, and all abnormalities were noted. All trachea and lung lobes were visually inspected and weighed, and any abnormal lesions were noted and described. The nasal turbinates and left lung with trachea of each animal were collected and these tissues were fixed with 10% neutral-buffered formalin for histopathological examination. Following fixation, tissue sections were embedded in paraffin blocks for processing, micro-sectioned, and stained with hematoxylin and eosin for histopathological examination by light microscopy. The following histopathological abnormalities were noted when present in the nasal turbinates or the lungs: severity of inflammatory cell infiltration, congestion, damage, edema, hemorrhage, hyperplasia/inflammation. All personnel performing the analyses in which interpretation of data was required were unaware of the study treatment assignments, and specimens were masked by allocating a unique sample number to each sample collected.

### Reporting summary

Further information on research design is available in the Nature Research Reporting Summary linked to this article.

## Supplementary information

Supplementary Material

Reporting Summary

## Data Availability

The data that support the findings of this study are available from the corresponding author upon request.
