# Supplementary material for: AS03-adjuvanted H7N9 inactivated split virion vaccines induce cross-reactive and protective responses in ferrets
Source: NPJ Vaccines. 2021 Mar 19;6:40. doi: 10.1038/s41541-021-00299-3 (PMC7979725; doi:10.1038/s41541-021-00299-3)
Supplement: Supplementary file 1 — Supplementary Material [file 41541_2021_299_MOESM1_ESM.pdf]

## Supplementary material

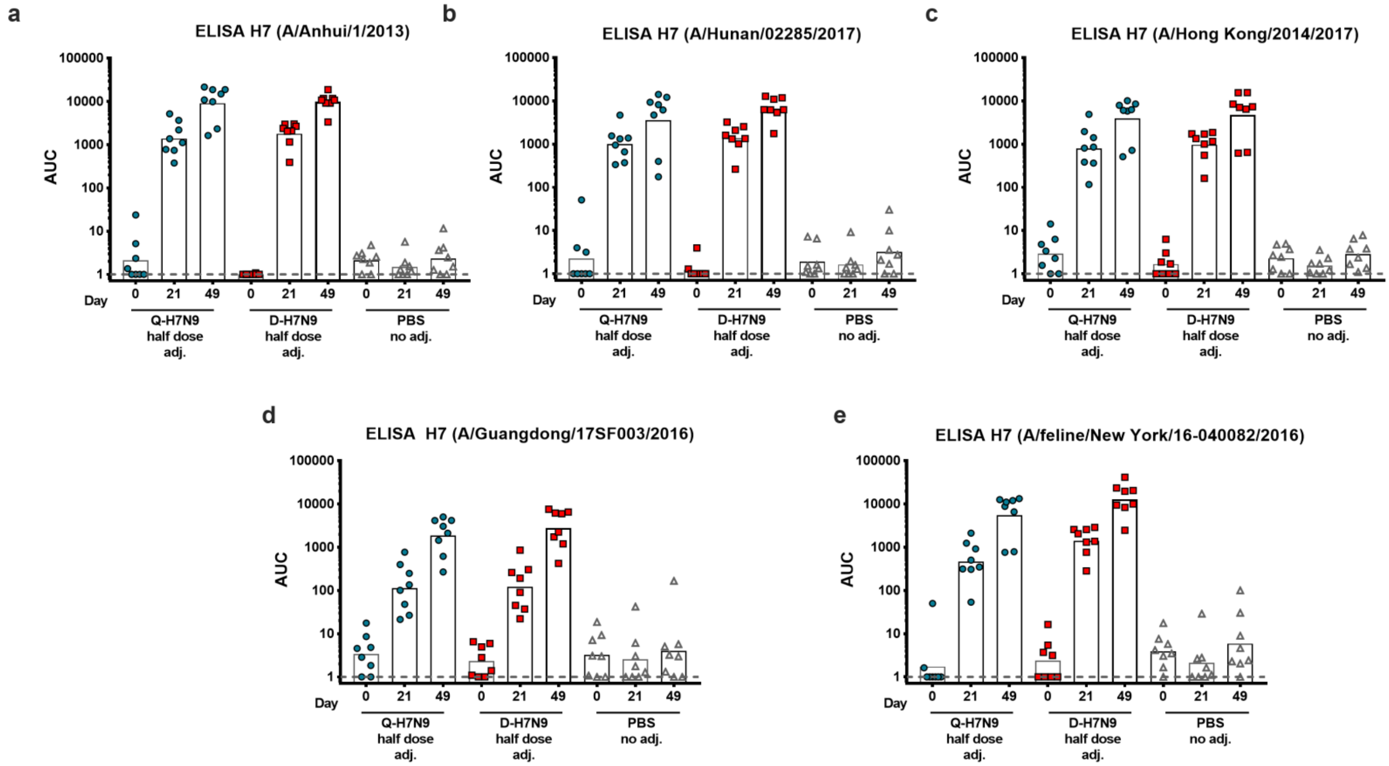


**Supplementary Figure 1. Cross-reactive antibody responses to HAs from emerging Eurasian and an American lineage H7 virus after H7N9 split virion immunization as measured by ELISA**. Serum samples from a sub-selection of samples (half dose HA + AS03 and PBS placebo group) were tested for binding to H7 HAs of different H7 viruses. Absolute ELISA area under the curve (AUC) values were determined for H7 HA of A/Anhui/1/2013 (**a**), A/Hunan/02285/2017 (**b**), A/Hong Kong/2014/2017 (**c**), A/Guangdong/17SF003/2016 (**d**) and A/feline/New York/16-040082-1/2016 (**e**). Data for baseline (day 0) and post-vaccination (day 21, day 49) serum samples are shown.

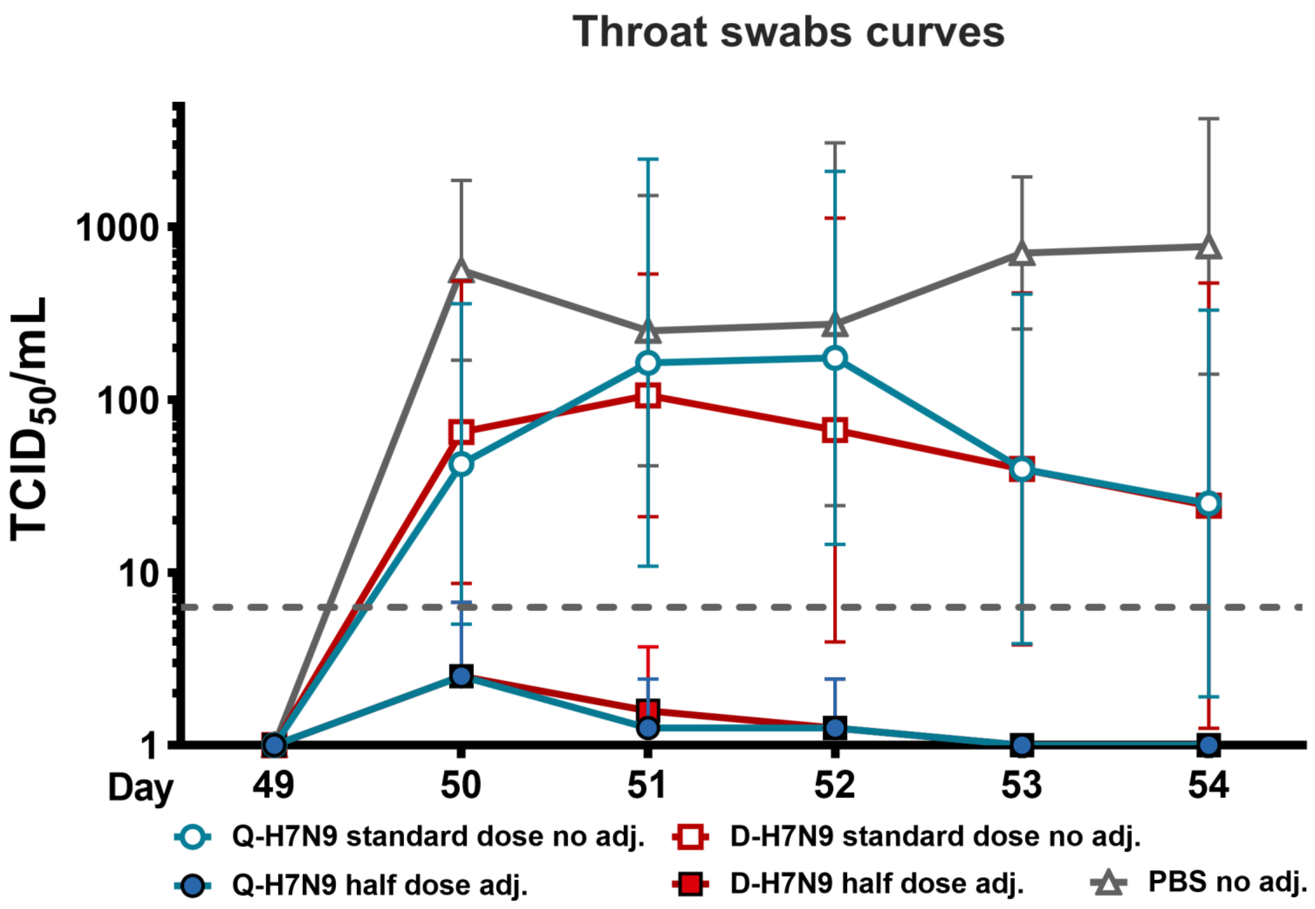


**Supplementary Figure 2. Throat swab curves.** Quantification of replication competent virus in throat swabs expressed as $TCID_{50}$/ml throat swab is depicted for ferrets receiving non- adjuvanted standard dose H7N9 split virion vaccine (open circles, open squares), AS03-adjuvanted half dose H7N9 split vaccine (closed circle, closed squares) and PBS (open triangle). The data are shown for day 0 until the day of euthanasia (day 5). The geometric mean of the $TCID_{50}$/mL and corresponding geometric standard deviation are shown.

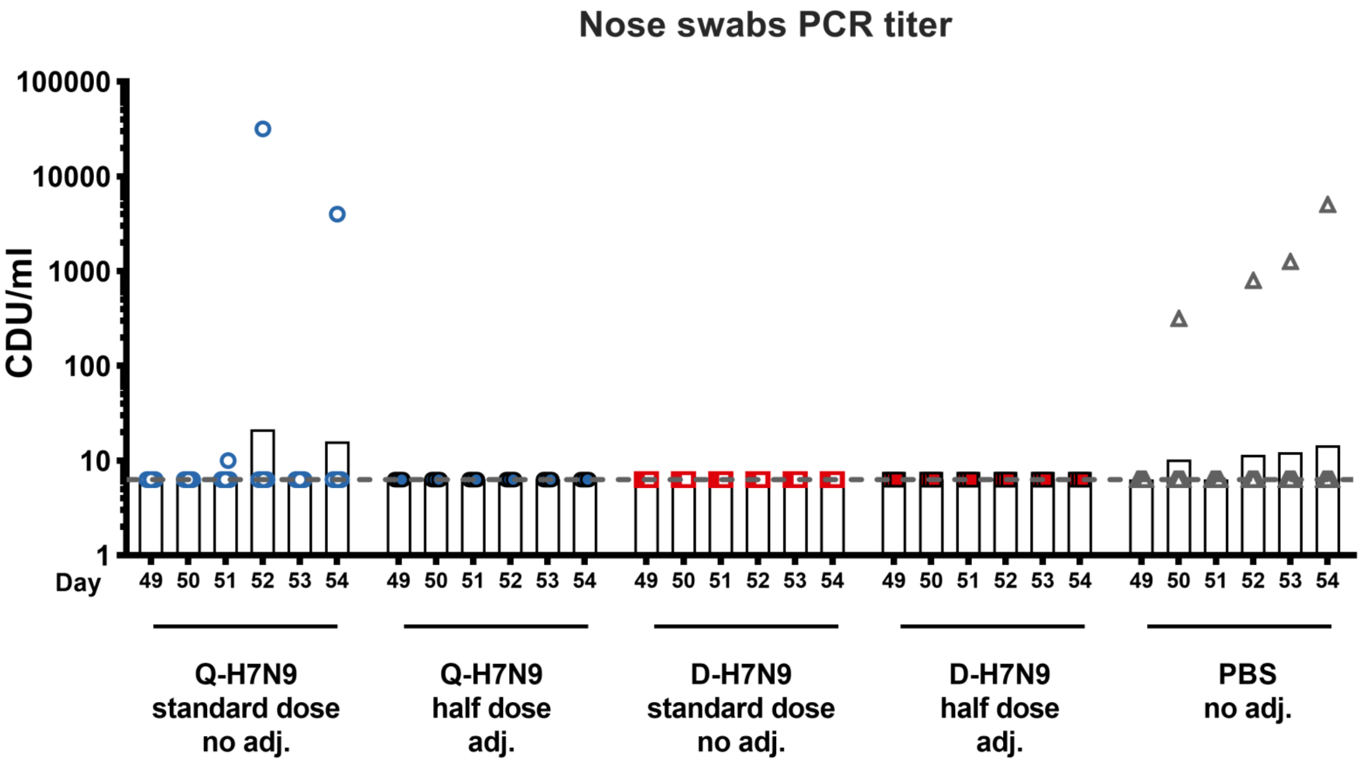


**Supplementary Figure 3. Nose swabs PCR titers.** Viral RNA in nose swabs were quantified by PCR. Viral RNA content is plotted on the y-axis as CDU/ml nose swab for day 0-5 post challenge for the five treatment groups.

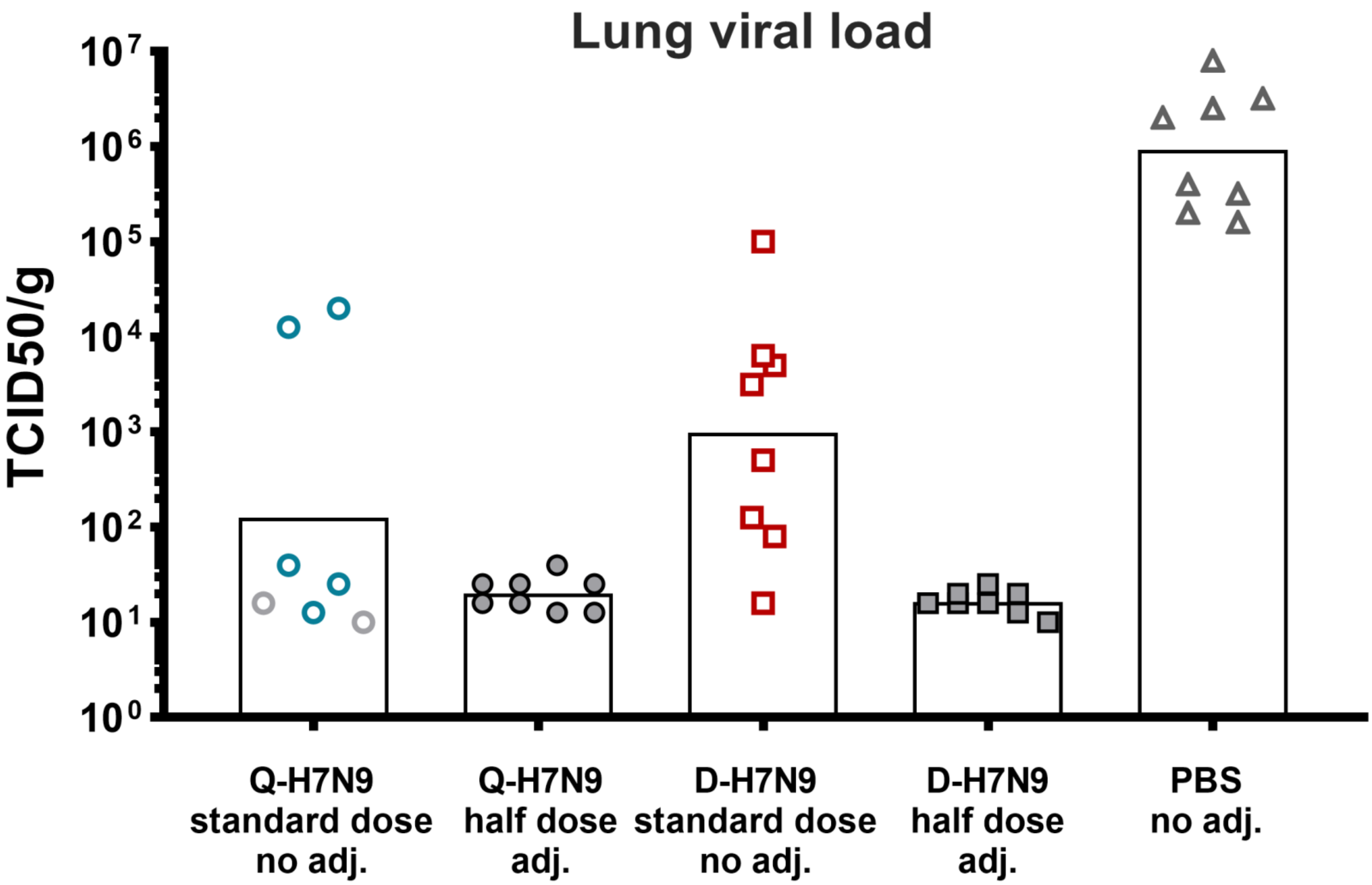


**Supplementary Figure 4. Viral load in lung tissue.** The lungs were harvested after euthanasia (day 5) and replication competent virus was quantified and recorded as $TCID_{50}$/g. The x-axis shows the five different experimental groups. Individual samples below the limit of detection are greyed out.

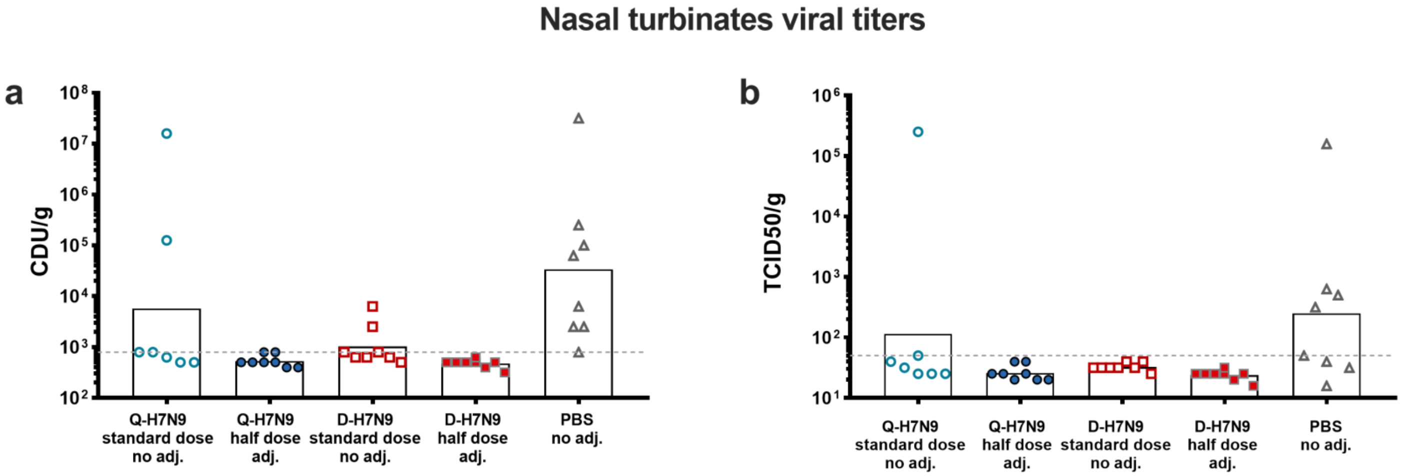


**Supplementary Figure 5. Viral load in nasal turbinates.** Levels of viral RNA in nasal turbinates were measured by means of PCR (**a**) and levels of replication competent virus were detected by virus titration on MDCK cells (**b**). Data are shown as CDU/g nasal turbinate and $TCID_{50}$/g nasal turbinate on the y-axis, respectively. The treatment groups are indicated on the x-axis. All samples with a value smaller than the value indicated by the dashed grey line fall below the limit of detection.

**Supplementary Table 1**. Amino acid identity between vaccine strain full-length H7 HA, H7 HA1 and H7 HA2 subunit and viruses used for virus challenge as well as serological assays.

| A/Shanghai/2/2013 | | H7 HA | | H7 HA1 | | H7 HA2 |
|---|---|---|---|---|---|---|
| A/Anhui1/2013 | H7 HA | 100% | H7 HA1 | 100% | H7 HA2 | 100% |
| A/Hong Kong/2014/2017 | H7 HA | 98.8% | H7 HA1 | 98.2% | H7 HA2 | 99.6% |
| A/Hunan/02285/2017 | H7 HA | 98.0% | H7 HA1 | 98.2% | H7 HA2 | 97.7% |
| A/Guangdong/17SF003/2016 | H7 HA | 97.3% | H7 HA1 | 96.2% | H7 HA2 | 98.6% |
| A/feline/New York/16-040082-1/2016 | H7 HA | 81.9% | H7 HA1 | 77.3% | H7 HA2 | 88.2% |
| A/Netherlands/219/2003 | H7 HA | 96.1% | H7 HA1 | 95.3% | H7 HA2 | 97.3% |
| A/mallard/Netherlands/12/2000 | H7 HA | 97.1% | H7 HA1 | 96.5% | H7 HA2 | 98.2% |

Amino acid sequences from the vaccine antigen was aligned to appropriate proteins from the influenza virus challenge strain and strains used for serological assays using the Clustal Omega multiple sequence alignment tool. Percent amino acid identity was determined using the computed percent identity matrix and examined for each virus used.
