# Supplementary material for: AS03-adjuvanted H7N9 inactivated split virion vaccines induce cross-reactive and protective responses in ferrets
Source: NPJ Vaccines. 2021 Mar 19;6:40. doi: 10.1038/s41541-021-00299-3 (PMC7979725; doi:10.1038/s41541-021-00299-3)
Supplement: Supplementary file 2 — Reporting Summary [file 41541_2021_299_MOESM2_ESM.pdf]

nature research

Corresponding author(s): Corey P. Mallett
Last updated by author(s): Dec 23, 2020

Reporting Summary

Nature Research wishes to improve the reproducibility of the work that we publish. This form provides structure for consistency and transparency in reporting. For further information on Nature Research policies, see our Editorial Policies and the Editorial Policy Checklist.

Statistics

For all statistical analyses, confirm that the following items are present in the figure legend, table legend, main text, or Methods section.

n/a	Confirmed	
☐	☒	The exact sample size (n) for each experimental group/condition, given as a discrete number and unit of measurement
☐	☒	A statement on whether measurements were taken from distinct samples or whether the same sample was measured repeatedly
☐	☒	The statistical test(s) used AND whether they are one- or two-sided<br>*Only common tests should be described solely by name; describe more complex techniques in the Methods section.*
☒	☐	A description of all covariates tested
☐	☒	A description of any assumptions or corrections, such as tests of normality and adjustment for multiple comparisons
☐	☒	A full description of the statistical parameters including central tendency (e.g. means) or other basic estimates (e.g. regression coefficient) AND variation (e.g. standard deviation) or associated estimates of uncertainty (e.g. confidence intervals)
☐	☒	For null hypothesis testing, the test statistic (e.g. F, t, r) with confidence intervals, effect sizes, degrees of freedom and P value noted<br>*Give P values as exact values whenever suitable.*
☒	☐	For Bayesian analysis, information on the choice of priors and Markov chain Monte Carlo settings
☒	☐	For hierarchical and complex designs, identification of the appropriate level for tests and full reporting of outcomes
☒	☐	Estimates of effect sizes (e.g. Cohen's d, Pearson's r), indicating how they were calculated

Our web collection on statistics for biologists contains articles on many of the points above.

Software and code

Policy information about availability of computer code

Data collection	Microsoft Excel
Data analysis	GraphPad Prism Version 8

For manuscripts utilizing custom algorithms or software that are central to the research but not yet described in published literature, software must be made available to editors and reviewers. We strongly encourage code deposition in a community repository (e.g. GitHub). See the Nature Research guidelines for submitting code & software for further information.

Data

Policy information about availability of data

All manuscripts must include a data availability statement. This statement should provide the following information, where applicable:

- Accession codes, unique identifiers, or web links for publicly available datasets
- A list of figures that have associated raw data
- A description of any restrictions on data availability

The data that support the findings of this study are available from the corresponding author upon request.

Field-specific reporting

Please select the one below that is the best fit for your research. If you are not sure, read the appropriate sections before making your selection.

☒ Life sciences ☐ Behavioural & social sciences ☐ Ecological, evolutionary & environmental sciences

For a reference copy of the document with all sections, see nature.com/documents/nr-reporting-summary-flat.pdf

Life sciences study design

All studies must disclose on these points even when the disclosure is negative.

Sample size	Animal (ferret) sample size per group (N=8) was determined by animal facility space restrictions and a scientifically "reasonable" number. Animals were challenge under BSL-3 containment and that influenced the samples size.
Data exclusions	No data were excluded.
Replication	Serum samples were analyzed by single determination and measurements were taken from distinct samples (as indicated in statistics section above).
Randomization	To obtain an equal distribution of body weights, the forty ferrets (total N for study) were divided among the study groups on the basis of their body weights using Excel for the randomization at the beginning of the study (Day 0).
Blinding	Personnel performing the histopathological analyses in which interpretation of data was required were unaware of the study treatment assignments.

Reporting for specific materials, systems and methods

We require information from authors about some types of materials, experimental systems and methods used in many studies. Here, indicate whether each material, system or method listed is relevant to your study. If you are not sure if a list item applies to your research, read the appropriate section before selecting a response.

Materials & experimental systems

n/a	Involved in the study	
☐	☒	Antibodies
☐	☒	Eukaryotic cell lines
☒	☐	Palaeontology and archaeology
☐	☒	Animals and other organisms
☒	☐	Human research participants
☒	☐	Clinical data
☒	☐	Dual use research of concern

Methods

n/a	Involved in the study	
☒	☐	ChIP-seq
☒	☐	Flow cytometry
☒	☐	MRI-based neuroimaging

Antibodies

Antibodies used	1. Ferret serum containing antibodies elicited by immunization with the H7N9 vaccines were tested in a variety of immunoassays (e.g., hemagglutination inhibition, microneutralization, and ELISA); 2. For the ELISA, goat anti-ferret IgG (gamma chain-specific) horseradish peroxidase (HRP) conjugate, Alpha Diagnostics, #70530 was used as the secondary antibody.
Validation	No validation was performed on the ferret serum antibodies, and the commercially-obtained anti-ferret IgG was tested for specificity by the manufacturer (Alpha Diagnostics).

Eukaryotic cell lines

Policy information about cell lines

Cell line source(s)	Madin Darby canine kidney (MDCK) cells, human embryonic kidney (293T) cells, and BTI-TN5B1-4 (Trichoplusia ni) cells were all obtained from ATCC.
Authentication	Cell lines were obtained from a commercial source (ATCC). After receipt, cells were recovered, passaged, and not further authenticated.
Mycoplasma contamination	Cell lines tested negative for mycoplasma contamination.

Commonly misidentified lines (See ICLAC register)	Commonly misidentified cell lines were not used.

Animals and other organisms

Policy information about studies involving animals; ARRIVE guidelines recommended for reporting animal research

Laboratory animals	Female ferrets (Mustela putorius furo) approximately 9 months of age at the beginning of the study (Day 0).
Wild animals	Wild animals were not used.
Field-collected samples	Field-collected samples were not used.
Ethics oversight	The animal study was carried out in the central animal facilities of The Netherlands Vaccine Institute (NVI; Bilthoven, The Netherlands). The NVI is approved for animal work under local legislation; Dutch Animal Experimentation Act, 1977 (Wet op de dierproeven, 1977), which is the Dutch equivalent to the European Council Directive 86/609 EEG. The protocol was licensed under protocol number 201300081 by the animal ethics committee. The study was conducted in accordance with the GSK Policy on the Care, Welfare and Treatment of Laboratory Animals and it was reviewed by the Institutional Animal Care and Use Committee at the NVI.

Note that full information on the approval of the study protocol must also be provided in the manuscript.
